# Subthreshold Oscillating Waves in Neural Tissue Propagate by Volume Conduction and Generate Interference

**DOI:** 10.3390/brainsci13010074

**Published:** 2022-12-30

**Authors:** Chia-Chu Chiang, Dominique M. Durand

**Affiliations:** Neural Engineering Center, Department of Biomedical Engineering, Case Western Reserve University, Cleveland, OH 44106, USA

**Keywords:** hippocampus, neural oscillation, optogenetics, propagation, subthreshold oscillation

## Abstract

Subthreshold neural oscillations have been observed in several brain regions and can influence the timing of neural spikes. However, the spatial extent and function of these spontaneous oscillations remain unclear. To study the mechanisms underlying these oscillations, we use optogenetic stimulation to generate oscillating waves in the longitudinal hippocampal slice expressing optopatch proteins. We found that optogenetic stimulation can generate two types of neural activity: suprathreshold neural spikes and subthreshold oscillating waves. Both waves could propagate bidirectionally at similar speeds and go through a transection of the tissue. The propagating speed is independent of the oscillating frequency but increases with increasing amplitudes of the waves. The endogenous electric fields generated by oscillating waves are about 0.6 mV/mm along the dendrites and about 0.3 mV/mm along the cell layer. We also observed that these oscillating waves could interfere with each other. Optical stimulation applied simultaneously at each slice end generated a larger wave in the middle of the tissue (constructive interference) or destructive interference with laser signals in opposite phase. However, the suprathreshold neural spikes were annihilated when they collided. Finally, the waves were not affected by the NMDA blocker (APV) and still propagated in the presence of tetrodotoxin (TTX) but at a significantly lower amplitude. The role of these subthreshold waves in neural function is unknown, but the results show that at low amplitude, the subthreshold propagating waves lack a refractory period allowing a novel analog form of preprocessing of neural activity by interference independent of synaptic transmission.

## 1. Introduction

Neural activity in the form of rhythmic or cycling oscillating patterns at specific frequency bands has been widely recorded in the whole brain, such as the slow oscillations in the cortex and hippocampus [1,2,3,4] and the theta waves in the hippocampus [5,6]. Slow oscillations are associated with memory consolidation [7] and theta waves are important for explorative behavior and cognition [8]. This oscillating neural activity is also observed to propagate across the brain and could play an important role in memory and spatial navigation [2,6,9]. In the hippocampus, propagating waves across the septal-temporal axis are thought to be a communication bridge to integrate the functions in different hippocampal regions [9,10,11].

Neural oscillations can be recorded as local field potentials in the neural tissue. In addition, at the cellular level, subthreshold oscillations are voltage changes of membrane potentials in a rhythmic pattern below the threshold for action potential generation. These subthreshold oscillations were first observed in the inferior olive [12]. Subthreshold oscillations at different frequency bands have also been observed in various areas across the brain, including entorhinal cortex [13,14], perirhinal cortex [15], frontal cortex [16,17], olfactory bulb [18], dorsal cochlear nucleus [19] and hippocampus [20,21,22,23]. Subthreshold oscillations can be observed alone or with superimposed action potentials. The function of subthreshold oscillations at the cellular level is still not clear, but they are thought to maintain a stable frequency at the neural network level [24] and modulate the timing of action potentials [25,26]. Other studies also propose that subthreshold oscillations can improve the communication of frequency-dependent properties across the network [27,28] and facilitate the encoding of the information [29]. 

In the hippocampus, neural oscillations at the neural network level and subthreshold oscillations at the cellular levels are found to be tightly involved with the timing of action potentials [22,23]. In particular, hippocampal theta activity could be a reflection of the underlying oscillating subthreshold membrane potentials [10,30,31,32]. However, subthreshold oscillations could also be generated by local endogenous field potentials. In the hippocampus, endogenous electric fields are known to underly waves propagating ephaptically (electric field coupling) at speeds similar to theta waves. Therefore, subthreshold oscillations could also be propagating waves sustained by endogenous fields. The determination of the causal role of extracellular electric fields on subthreshold oscillations is difficult to assess since stimulation artifacts can confound the result. To circumvent the electrical stimulation artifact and study the spatial extent and propagation of subthreshold oscillations, we generated neural oscillations optically using the optogenetic technique [33]. In this manuscript, we apply optogenetic stimulation to trigger subthreshold waves at various frequencies and study the propagation and characteristics of these waves in the hippocampus. 

## 2. Results

### 2.1. Optogenetic Stimulation Can Trigger Propagating Subthreshold Oscillating Waves at Various Frequencies and Suprathreshold Neural Spikes in the Hippocampal Slice

**Figure 1:**
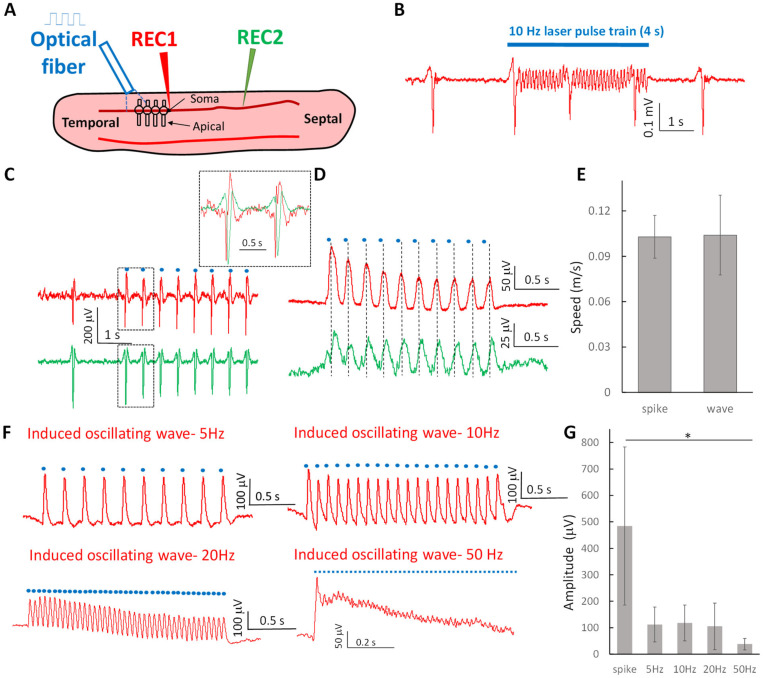
Neural activity induced by optogenetic stimulation in the longitudinal hippocampal slice. (**A**) The experimental setup to trigger neural activity by optogenetic stimulation. Two electrodes were positioned on the cellular layer and an optic fiber was placed near the recording electrodes to deliver the laser pulse train. (**B**) The laser pulse train at 10 Hz can trigger two types of neural activity. One was the suprathreshold spikes with an amplitude similar to that of spontaneous spikes. The other was an oscillating wave with a smaller amplitude. (**C**) The optogenetic induced spikes by single laser pulse can propagate through the longitudinal slice. The expanded window shows that there are delays between two spikes recorded from REC1 and REC2 electrodes. (**D**) Similarly, two oscillating waves triggered by 5 Hz laser pulse train and recorded from REC1 and REC2 electrodes have delays in each cycle. (**E**) The speeds of the spikes and oscillating waves are not significantly different. (**F**) 5 Hz pulse train can trigger a 5 Hz oscillating wave. The wave circles followed the laser pulse train. Similarly, 10 Hz, 20 Hz, and 50 Hz pulse trains can trigger 10 Hz, 20 Hz, and 50 Hz oscillating waves individually. (**G**) The amplitude of the induced spikes was higher than those of the oscillating waves at 5, 10, 20, and 50 Hz (*: *p* < 0.05, *n* = 6 slices).

Neural oscillations have been generated in the form of spontaneous slow periodic spikes in the in vitro cortical and hippocampal slices by lowering the ionic concentration of calcium and magnesium [34,35,36]. To study the propagation of these oscillating waves, it is advantageous to stimulate the activity in order to control the amplitude and frequency. Thus, optogenetic stimulation is preferred to eliminate the stimulation artifacts. To determine whether optogenetic stimulation can generate oscillating waves, a 10 Hz laser pulse train was applied to the longitudinal hippocampal slices expressing optopatch in Camk2a^+^ neurons [33] perfused with the aCSF with lowered concentrations of calcium and magnesium (Figure 1A). Two types of activity were recorded from the cell layer of longitudinal hippocampus slices: high-amplitude spikes (484 ± 298 μV) and small amplitude oscillating waves (117 ± 68 μV) (Figure 1B). The spikes were often triggered at the onset of laser activation pulse or appeared spontaneously during the optical stimulation window (Figure 1B,C). Both spikes induced by a single pulse and oscillating waves induced by a pulse train in the temporal regions were observed to propagate through the slice (Figure 1C,D). The speeds of these two activities were similar (Figure 1E) and the average speeds of the spikes and waves were 0.10 ± 0.02 m/s and 0.11 ± 0.03 m/s, respectively. The triggered oscillating wave followed a series of on-off signal of the laser pulse and, therefore, the frequency of the wave could be controlled by the laser pulse train to mimic the neural activity oscillating in different frequency recoded in the tissue. Examples of waves recorded at 5, 10, 20 and 50 Hz are shown in Figure 1F. The amplitudes of oscillating waves decreased with increasing frequency (112 ± 66 μV at 5 Hz, 117 ± 68 μV at 10 Hz, 105 ± 88 μV at 20 Hz, and 37 ± 22 μV at 50 Hz) and were significantly smaller than that of the spikes (484 ± 298 μV, *p* < 0.05, *n* = 6 slices) (Figure 1G). Therefore, these results show that the laser-induced propagating oscillating waves are below the threshold for activating spikes. The induced high-amplitude spikes are similar to previously recorded spontaneous activity [34,36]. To confirm that the waves were generated by neurons in response to the laser pulse, the laser was also applied to a slice from the wild-type mice but was no response to the laser pulse was observed (*n* = 2). 

### 2.2. Oscillating Wave Propagate by Volume Conduction through Electric Field Coupling

**Figure 2:**
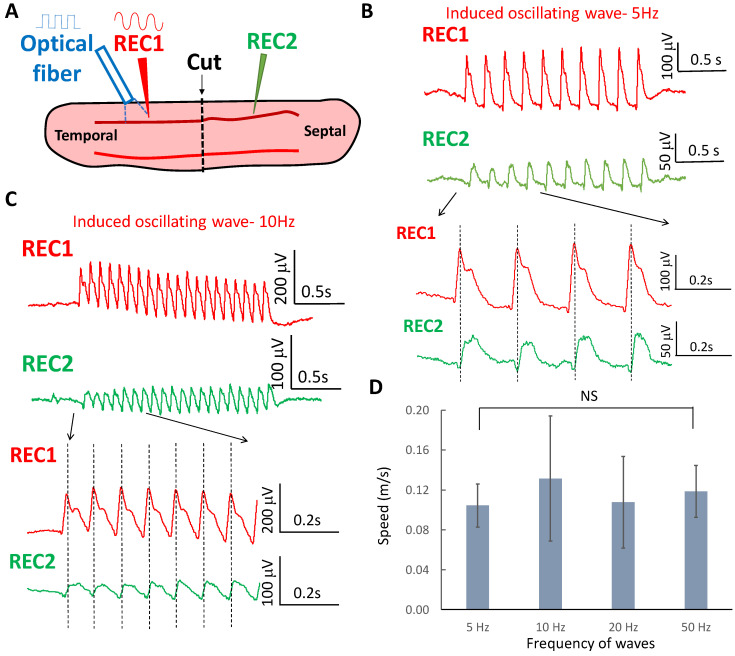
Propagation of subthreshold wave across the transection in the slice. (**A**) A transection was made in the middle of the slice. Two electrodes (REC1 and REC2) were placed on both sides of the slice to monitor the propagation. The laser pulse train was applied on one side of the slice to initiate the waves. (**B**) The 5 Hz wave can cross the transection while the subthreshold wave can be recorded in both electrodes with the presence of the transection. The expanded window shows that the delay was observed between two waves from REC1 and REC2. (**C**) The 10 Hz wave can propagate through the transection. The expanded window shows the wave can cross the transection with a delay. (**D**) The speeds of the waves at various frequency bands were similar across the frequency (*n* = 6 slices).

**Figure 3:**
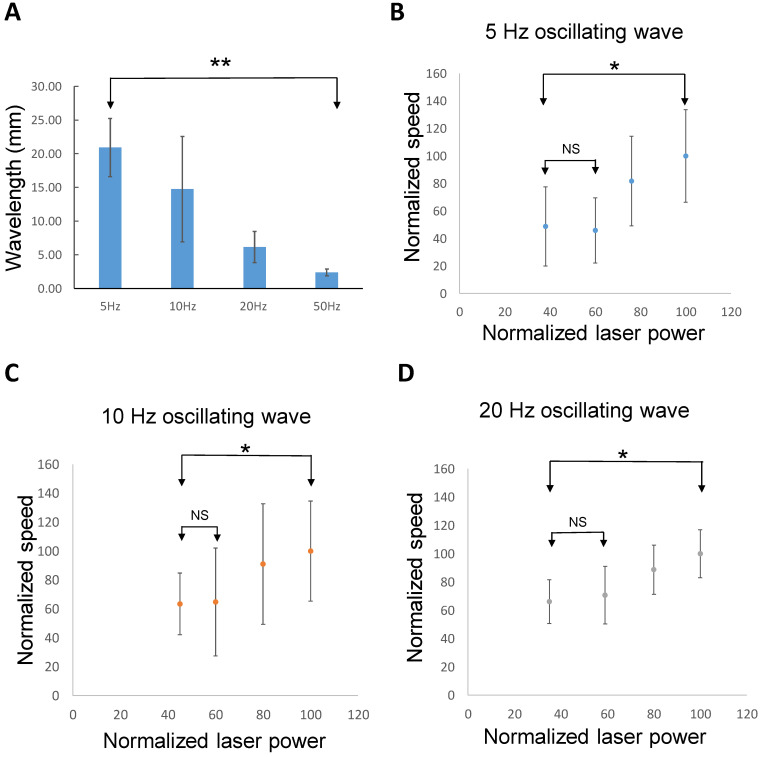
The wavelength and amplitude-dependent propagation of the subthreshold waves. (**A**) The wavelength of the subthreshold waves decreased with frequency (**: *p* < 0.01, *n* = 6 slices). (**B**) The speed of the subthreshold wave decreased when the amplitude of the waves reduced (*: *p* < 0.05, *n* = 5 slices). (**C**) The speed decayed with the amplitude drop at 10 Hz subthreshold waves (*: *p* < 0.05, *n* = 5 slices). (**D**) A similar trend was also observed in the 20 Hz subthreshold waves (*: *p* < 0.05, *n* = 5 slices).

It has been shown that suprathreshold spikes both in the cortex and hippocampus can propagate across the transection between two synaptically disconnected brain slices [35,36]. The speed of the laser-evoked subthreshold oscillating waves suggests that they could also spread without synaptic transmission through electric field (ephaptic) coupling [37,38]. To determine whether oscillating waves in different frequency range could also propagate without synaptic transmission, two recording electrodes were placed on both sides of the hippocampal slice and a complete transection of the tissue was carried out between the two electrodes. The laser was then applied to one side to trigger the oscillating waves (Figure 2A). After transection was made in the slice, induced oscillating waves at 5, 10, 20, and 50 Hz were observed to propagate from one end to the other of the slice through the cut. The wave and propagation delay at 5 and 10 Hz are shown in Figure 2B,C, respectively. The propagating speed of the oscillating wave was 0.10 ± 0.02 m/s at 5 Hz, 0.13 ± 0.06 m/s at 10 Hz, 0.11 ± 0.06 m/s at 20 Hz, and 0.12 ± 0.03 m/s at 50 Hz (*n* = 6 slices). The speeds of the oscillating waves were not significantly different across the various frequencies of stimulation (Figure 2D). However, the wavelength of the oscillating waves decreased with the frequency (*p* < 0.01, *n* = 6 slices) (Figure 3A). The range of the wavelength of the oscillating wave was 20.9 ± 4.3 mm at 5 Hz, 14.7 ± 7.8 mm at 10 Hz, 6.1 ± 2.3 mm at 20 Hz, and 2.4 ± 0.5 mm at 50 Hz (*n* = 6 slices). 

It has been shown that the speed of propagation through ephaptic coupling is related to the electric field amplitude [37]. To determine the relationship between the speed and the amplitude of the oscillating waves, the laser pulse intensity was set at different levels and the speeds of propagation were measured. When the laser intensity was set below 60%, the speed of the oscillating wave remained similar (Figure 3C,D). However, as the intensity of the laser increased, the speeds of the oscillating waves at 5, 10, and 20 Hz increased proportionally (*p* < 0.05, *n* = 5 slices).

### 2.3. Propagating Oscillating Waves Generate Very Low Amplitude Electric Fields

**Figure 4:**
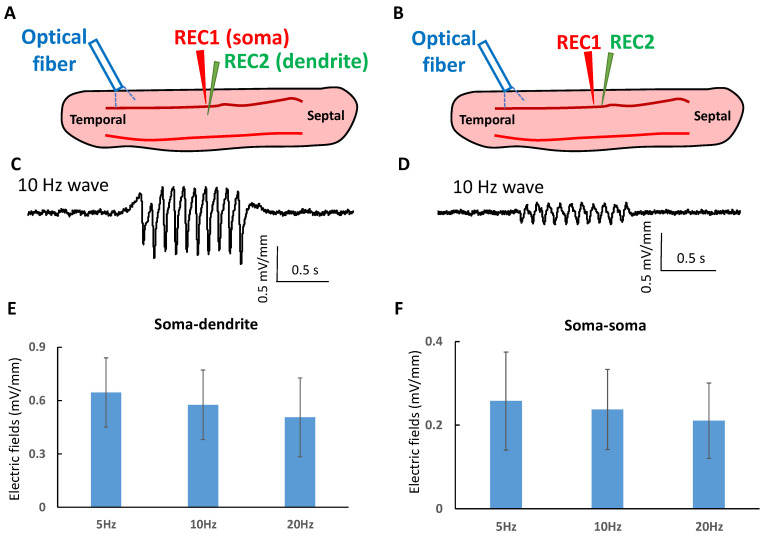
The endogenous electric fields of the subthreshold waves. (**A**) The electric field measurement between somatic and dendritic layers. Two electrodes were placed in the somatic and dendritic layers distal to the initiation of the wave to measure the voltage difference. (**B**) The field measurement along the longitudinal direction. Two electrodes were placed in the cellular layer to measure the voltage and calculate the electric field. (**C**) An example of the measured electric field between somatic and dendritic layers when the 10 Hz wave was present. (**D**) An example of the electric field in the longitudinal direction at 10 Hz wave. (**E**) The electric fields between somatic and dendritic layers were measured at 5 Hz, 10 Hz, and 20 Hz waves (*n* = 5 slices). (**F**) The electric fields along the longitudinal direction were measured at 5 Hz, 10 Hz, and 20 Hz waves (*n* = 5 slices).

The wave is initiated by the laser but propagates into other region of the slice through the transection. Therefore, the propagation could be sustained by endogenous fields as previously observed in other studies with suprathreshold spiking activity [35,39]. The amplitudes of endogenous electric fields generated by the propagating waves were measured in the tissue at different orientations. To measure the electric field perpendicular to the direction of propagation (E_P_), two electrodes were placed in the somatic and dendritic layer. The voltage difference was measured and divided by the distance between electrodes (Figure 4A). Similarly, the electric fields in the longitudinal direction (E_L_) parallel to the direction of propagation were estimated by placing two electrodes along the cell layer (Figure 4B). Examples of the waveforms for E_P_ and E_L_ obtained for 10 Hz stimulation are shown in Figure 4C,D. The peak to peak amplitudes of the measured electric fields when the oscillating waves at 5, 10, and 20 Hz were 0.65 ± 0.19 mV/mm, 0.57 ± 0.20 mV/mm, and 0.51 ± 0.22 mV/mm individually (Figure 4E, *n* = 5 slices). No statistical difference between these amplitudes was detected and therefore, these results show that within a range of 5 to 20 Hz, the velocity of the propagation is independent of frequency (*p* > 0.5) (Figure 2D). Electric fields in the longitudinal direction were found to be 0.26 ± 0.11 mV/mm, 0.24 ± 0.09 mV/mm, and 0.21 ± 0.09 mV/mm at 5, 10, and 20 Hz, respectively (Figure 4F, *n* = 5 slices). The longitudinal electric fields were significantly smaller than the perpendicular fields (*p* < 0.01 at 5 Hz, *p* < 0.01 at 10 Hz, and *p* < 0.05 at 20 Hz) and the speed was also independent of frequency in the same frequency range. The electric field amplitudes generated by these waves are well below the 1 mV/mm threshold for neural membrane polarization by applied fields [40]. 

### 2.4. Oscillating Waves Interfere with Each Other Constructively and Destructively

**Figure 5:**
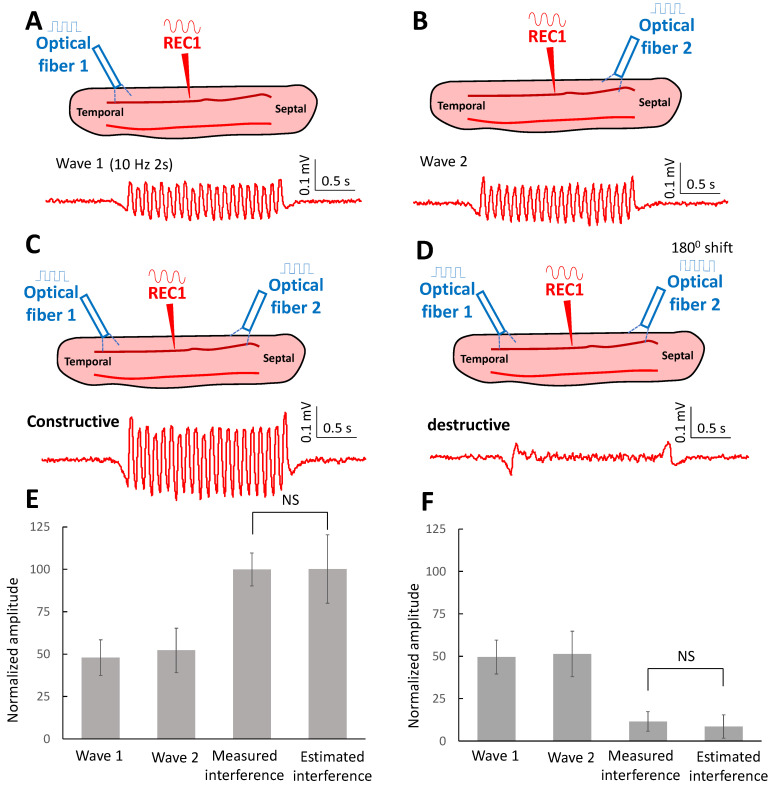
Interference of the subthreshold waves. (**A**) The subthreshold wave at 10 Hz was triggered in the temporal region of the slice and was recorded in the middle of the slice. (**B**) Similarly, the subthreshold wave can be triggered in the septal area and recorded in the middle of the slice. (**C**) When the laser was applied on both sides of the slice and generated two subthreshold with the same phase, larger subthreshold waves were recorded in the middle of the slice. (**D**) When the laser triggered two waves with a 180-degree phase shift, a smaller subthreshold wave was observed in the middle of the slice. (**E**) The amplitude of subthreshold waves reconstructed by adding two encountering waves was similar to that measured from the recording signal. It shows that the waves can constructively interfere with each other (*n* = 5 slices). (**F**) The amplitude of subthreshold waves from the estimated waves was similar to that from the recorded waves. It shows that the waves can also destructively interfere with each other (*n* = 5 slices).

The low amplitude of these waves and the lack of associated superthreshold spikes suggests that the waves are subthreshold and do not generate a refractory response in the neurons, thereby allowing interference. To test this hypothesis, we placed two lasers at each end of the tissue slice. To determine if the waves can interfere constructively, 10 Hz waves were triggered either from the temporal region of the slice (Figure 5A) or the septal area (Figure 5B) and the recording electrode was placed at a location equidistant from the two laser excitation regions to monitor the amplitude of the activity. When the laser pulse targeted both ends of the slice simultaneously, a higher amplitude of the oscillating wave was recorded (Figure 5C). The amplitude recorded in the middle of the slice was compared to the sum of the two incoming waves (estimated amplitude). The estimated amplitude of the oscillating wave was similar to the amplitude from the recorded waves triggered on both ends of the slice and no statistical difference was found (*p* > 0.5, *n* = 5 slices). The amplitude of the recorded oscillating wave was 100 ± 9.7%, and the estimated amplitude was 100 ± 20.1%. 

We then tested the hypothesis that destructive interference could also be generated. The lasers at each end of the slice were set to generate stimulation waveforms with 180° phase shift. A smaller amplitude of the wave was recorded in the middle of the slice (Figure 5D). The amplitude analysis shows that the estimated amplitude was similar to the amplitude of the recorded oscillating waves while the estimated amplitude was 8.5 ± 6.9% and the recorded amplitude was 11.5 ± 5.7% (*n* = 5 slices). These results suggest that these low amplitudes propagating waves are indeed subthreshold and can interfere both constructively and destructively with each other. 

**Figure 6:**
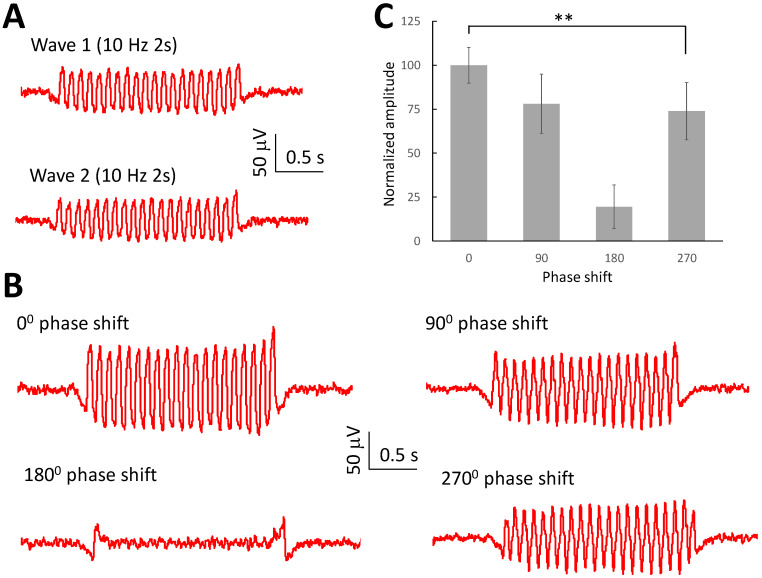
Interference effect related to the phase shift between two waves. (**A**) Waves were initiated at either the temporal area or septal area. (**B**) Interference between two waves was observed in the middle area of the slice with a phase shift of 0, 90, 180, and 270 degrees between the two waves. (**C**) The amplitude of the interference between the waves with a phase shift between two waves (**: *p* < 0.01, *n* = 6 slices).

Since the oscillating waves can interfere with each other, we further studied the effect when with different phase angles. Therefore, similar experiments were conducted to initiate two oscillating waves in the temporal or septal areas (Figure 6A). The interfered wave reached the maximum amplitude when two waves were in phase (0° phase shift). When two waves were slightly out of phase with 90° or 270° phase shift, the amplitude of the interfered wave dropped and reached a minimum value when the phase shift was 180° (Figure 6B). The amplitude analysis shows that the amplitude of the wave changed with the phase shift between two waves (*p* < 0.01, *n* = 6 slices). 

### 2.5. Suprathreshold Neural Spikes Collide When Encountering Other Spikes

**Figure 7:**
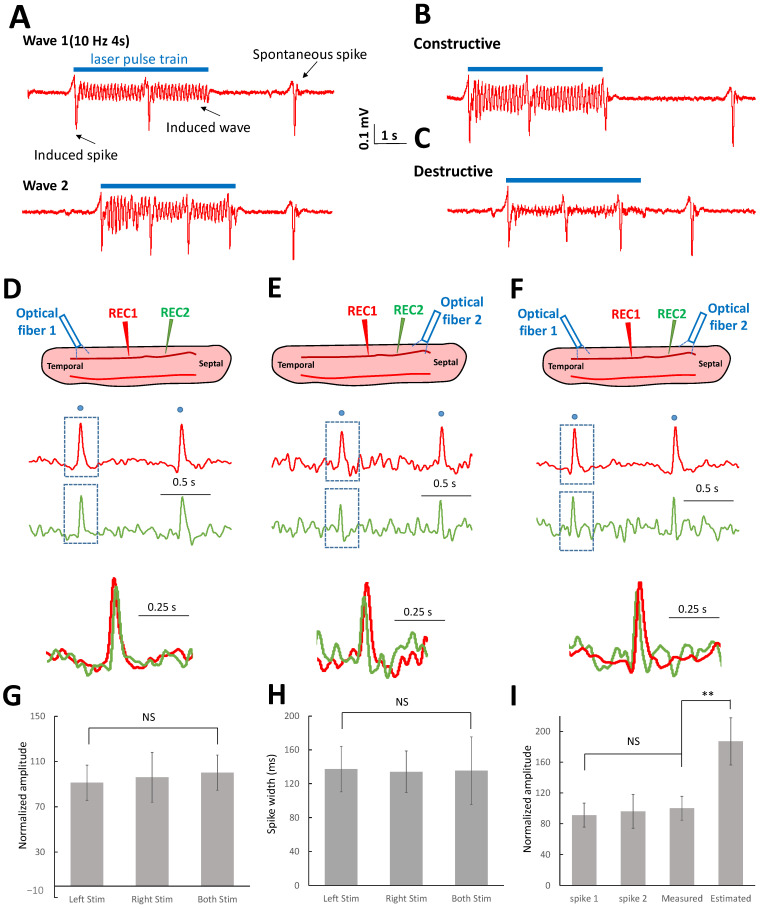
Collision of the suprathreshold spikes. (**A**) Both suprathreshold spikes and subthreshold waves were triggered in the temporal area of the slice or initiated in the septal area of the slice. (**B**) The constructive interference can only be observed outside the window of the suprathreshold spike duration. (**C**) Similarly, destructive interference can only happen without the presence of the suprathreshold spikes. (**D**) The suprathreshold spike was triggered in the temporal region of the slice and propagated to the septal region. (**E**) Similarly, the suprathreshold spikes can be triggered in the septal area and traveled to the temporal area. (**F**) When the laser was applied on both sides of the slice and generated suprathreshold spikes simultaneously, only one spike was recorded through the slice instead of two spikes. (**G**) The normalized amplitudes of the spikes triggered by the laser from the left side, right side, or both sides were not significantly different. (**H**) The pulse widths of the spikes triggered by the laser from the left side, right side, or both sides were similar. (**I**) By analyzing the amplitudes from the signals in each condition, the amplitudes of these suprathreshold spikes were similar. However, the estimated amplitude was higher than the real amplitude of the spike when assuming the spikes can interfere with each other (**: *p* < 0.05, *n* = 3 slices).

The laser pulses could trigger both spontaneous large amplitude spikes superimposed on the low amplitude oscillating waves (Figure 1B). Both the spikes and waves were observed to propagate by targeting either end of the slice with a laser (Figure 1C,D). In the presence of the spikes, the interference of the oscillating waves could still be observed (Figure 7A–C) between the occurrence of spiking activity. However, spike activity did not produce any interference patterns that interfered with each other as they were not observed to summate or subtract (Figure 7B,C). We then tested the hypothesis that suprathreshold spikes would annihilate each other during collision.

To test the collision hypothesis between two spikes travelling in opposite directions, a laser pulse was applied on the temporal side, septal side, or both sides simultaneously (Figure 7D–F). The laser pulse could successfully trigger the spikes on either side of the slice, and the spikes could propagate to the other side (Figure 7D,E). When laser pulses were simultaneously applied on both sides of the slices, triggered spikes were expected to come from both sides and thus two spikes should be recorded. However, the experiments show that only one spike was recorded after applying the laser pulses (Figure 7F), implying the spikes collided when encountered. The amplitudes and pulse widths of the spikes triggered from either one side of stimulation and both sides of stimulation were similar (Figure 7G,H). Moreover, assuming constructive interference on the spike, the estimated amplitude of the spikes was higher than the recorded spike (Figure 7I, *p* < 0.01). These analyses show that suprathreshold spikes cannot interfere with each other but collide in the middle of the slice. 

### 2.6. Oscillating Waves Are Independent of NMDA Receptors

**Figure 8:**
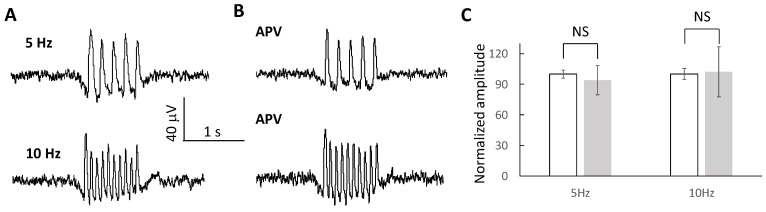
NMDA receptor properties of the subthreshold waves. (**A**,**B**) The subthreshold waves can be triggered at 5 Hz and 10 Hz before and after the application of the NMDA receptor blocker, APV. (**C**) The amplitudes of the subthreshold waves were similar with and without the presence of APV (*n* = 5 slices).

It has been shown that the NMDA receptor antagonist ((2R)-amino-5-phosphonovaleric acid; APV) can greatly reduce the amplitude of the suprathreshold spike generated in the preparation of the present study [34,36]. Therefore, NMDA receptors could be required to generate the oscillating waves triggered by the optogenetic stimulation. To test this hypothesis, laser pulses at 5 and 10 Hz were applied before and after the hippocampal slice was perfused with 50 μM APV. The oscillating waves at 5 and 10 Hz can be triggered under both conditions (Figure 8A,B). The amplitudes of the oscillating waves before the application of APV were 100 ± 3.9% and 100 ± 5.4% at 5 Hz and 10 Hz, respectively. Following the application of APV, the amplitudes of the oscillating waves were 94 ± 14.3% and 102 ± 24.4% at 5 Hz and 10 Hz, respectively (*n* = 5 slices). Therefore, the amplitudes of the waves were not affected by the application of APV (Figure 8C). 

### 2.7. Subthreshold Oscillating Waves Are Modulated by Sodium Channels

**Figure 9:**
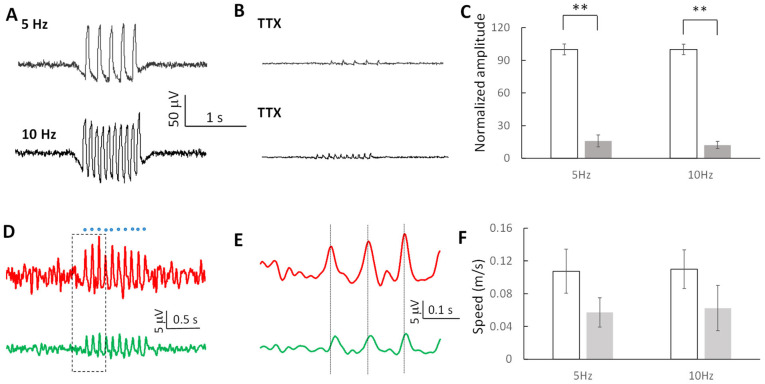
Sodium channel properties of the subthreshold waves. (**A**,**B**) Subthreshold waves at 5 Hz and 10 Hz can be triggered before the application of TTX but the amplitude was greatly reduced following the application of TTX. (**C**) Amplitudes of the subthreshold waves decreased after the slice was perfused with TTX solution (**: *p* < 0.01, *n* = 5 slices). (**D**) Induced oscillating wave can still propagate through the slice with smaller amplitude after the application of TTX. (**E**) Expanded window of (**D**) show the delays between two waves from different electrodes. (**F**) Speeds of the oscillating waves at both 5 Hz and 10 Hz decreased after the blocking the sodium channels (*n* = 5 slices).

To determine whether the induced oscillating waves were sodium-channel-dependent, a common voltage-gated channel in neuronal cells, optogenetic stimulation was applied before and following perfusion with 1 μM tetrodotoxin (TTX). Although wave propagation could still be observed in the presence of TTX, both the amplitude and the speed of oscillating waves were significantly reduced (Figure 9A,B). The amplitudes of the waves dropped to 15.9 ± 5.4% and 12.2 ± 3.3% of the amplitude before TTX application at 5 Hz and 10 Hz, respectively (*n* = 5 slices) and were significantly lower (*p* < 0.01, Figure 9C). Surprisingly, although the amplitude of the oscillating waves was significantly reduced by about 86% by TTX, the waves were still observed to propagate (Figure 9D,E). However, the speeds of the waves decreased by 47% and 44% in 5 Hz and 10 Hz waves, respectively, in the presence of TTX (Figure 9F). Taken together, these results suggest that small extracellular amplitude, TTX-sensitive subthreshold waves can propagate by electric field coupling and generate interference whereas superthreshold spikes do not interfere but instead are annihilated during the collision.

## 3. Discussion

The hippocampus exhibits functional spatial differentiation along the septotemporal axis. The septal hippocampus plays a major role in emotional memory, while the temporal hippocampus is more responsible for spatial memory [41,42,43]. Therefore, the propagating theta wave in the hippocampus has been proposed as a means of integration of the information contents between these two areas [9,10,11]. Theta activity at the network level has been observed to propagate at the same speed reported here [10] and thus theta activity could be a reflection of a subthreshold oscillation spreading across a neuronal population [10,30,31]. The subthreshold waves in the present study can propagate with wavelengths consistent with those observed in the rodent hippocampus [10] or the surface of the human hippocampus [11]. The spatial wavelength of these oscillating waves is in the millimeter range and the rodent hippocampus is several millimeters long. Therefore, a subthreshold wave propagating with a frequency in the theta range and wavelength of about 14 mm (Figure 3A), will encompass the entire rodent hippocampus. Furthermore, these subthreshold waves can propagate non-synaptic ally across the hippocampal slice at various frequency bands with similar speeds (Figure 2D). A propagation speed independent of frequency ensures minimum distortion during propagation and therefore, implies that there exists in the hippocampus a mechanism that allows analog propagation of neural activity along the hippocampus that is both distortion-free within a narrow frequency range and independent of synaptic transmission.

Neural activity can travel in the brain as a wave propagating in multiple directions [11,44,45] and is important in memory processing [45]. Studies show that traveling theta and alpha waves can propagate in posterior-to-anterior and anterior-to-posterior directions, respectively, during encoding and retrieval processes [45]. The present study shows that non-synaptic (ephaptic) subthreshold waves can be triggered on either side of the hippocampal slice and propagate across the transection through electric field coupling to other regions. Therefore, ephaptic subthreshold waves could play an important role in the memory process, similar to the traveling waves recorded in other studies. Conversely, the results suggest that theta waves could themselves be subthreshold waves propagating by volume conduction. A recent study also showed that 4-aminopyridine induced theta wave could cross a complete transection of the hippocampus in vivo [46]. 

The triggered subthreshold waves were resistant to NMDA receptor blocker, which has been shown to suppress the suprathreshold spikes in this preparation and in other experiments [34,36]. This implies that subthreshold waves are distinct from the suprathreshold spikes and have different underlying mechanisms. In addition, subthreshold waves were suppressed greatly by the sodium channel blocker, TTX, which suggests that sodium channels play a significant role in amplifying the subthreshold wave. It has been shown that subthreshold theta-like oscillations were sodium-dependent and relied on the persistent sodium channel [13,24]. These studies support the hypothesis that subthreshold waves require persistent sodium channel activity to maintain signal amplitude during propagation. A residual amplitude of the subthreshold waves was still present in the presence of TTX, suggesting that the induced oscillating wave can be under threshold of sodium channels. This TTX-independent propagating activity of low amplitude oscillations was also observed in cortical slices [35].

Intrinsic subthreshold membrane potential oscillations (STOs) refer to a rhythmic neural activity recorded intracellularly [13,14,20,21]. Even though the subthreshold waves in the present study were local field potentials, they share many properties with STOs. The subthreshold waves are low amplitude oscillating activity (Figure 1G) and the STOs oscillate with a low amplitude below the threshold of the spike [20,21,47]. Subthreshold waves are not affected by NMDA blocker APV (Figure 8) and STOs are also resistant to APV [47]. STOs can be suppressed by sodium channel blocker TTX [13,24,47], a result similar to that reported here for subthreshold waves (Figure 9). Moreover, both subthreshold waves and STOs can co-exist with the suprathreshold spikes [47]. Therefore, the subthreshold waves in the present study have distinct dynamics compared to the suprathreshold spike but share similar characteristics with STOs. 

Subthreshold waves can propagate through the hippocampal slice generating extracellular electric fields smaller than 1 mV/mm. It has been shown that neural activity can be modulated by the endogenous electric fields or applied electric fields [35,48]. Moreover, it was found that theta and alpha waves can be modulated by transcranial electric stimulation [49,50]. Transcranial electric stimulation is known to generate electric fields in the brain as low as 0.4 mV/mm [51]. It is therefore possible that the electric fields generated by transcranial electric stimulation could modulate existing ephaptic subthreshold waves thereby explaining some of the effects of transcranial electric stimulation. 

The present study also shows that subthreshold waves can interfere with each other. This is a highly surprising and novel finding as the refractory period associated with neural processing involving action potentials prevents interference. A possible explanation is that the neurons involved in producing the subthreshold waves do not reach refractory period at these very low amplitudes. This is supported by the fact that the sodium persistent channel, a subthreshold channel known to be involved in these waves have little or no inactivation [28,52] and therefore no significant refractory period. Therefore, these subthreshold waves are oscillating membrane potentials propagating below the threshold levels for action potentials. The situation is clearly different for the spikes, which are clearly suprathreshold and annihilated each other when colliding, a clear indication that there is a refractory period associated with each spike. 

One the major finding of this study is that neural signals can interfere with each other and generate both constructive and destructive interference. This interference phenomenon must be distinguished from the oscillatory interference model proposed to explain the regular spatial firing pattern in the grid cell [53]. This oscillatory interference model proposes that there are theta oscillators with various movement directions in the entorhinal cortex. The spatial interference between these oscillators causes grid cells to fire when they are in the constructive phase, thereby producing a spatial firing pattern [54,55]. These theta oscillators are thought to be traveling waves recorded at the neural network level [9,56], or intrinsic subthreshold membrane potential oscillations in single neurons of the entorhinal cortex [13,14]. The interference of subthreshold waves reported here is a different phenomenon from the oscillatory model, since signals are at different phases in order to generate interference patterns. We report above the results of experiments showing constructive and destructive interference of two waveforms at the same frequency. This can be explained by the fact that the mechanism underlying the propagation involves subthreshold channels with no refractory period. Another possible theory to explain the propagation of neural waves in the brain is the soliton wave mechanism [57]. However, the soliton wave equations cannot explain the results presented in this study since soliton theory predicts that when two waves pass through each other without summation or collision [57]. The results here show that subthreshold waves interfere in the standard wave theory and suprathreshold spikes collide.

Although neural waves and oscillations can be generated spontaneously, analysis of the interference pattern requires that the timing, phase and direction be controlled. Electrical stimulation is a possible mechanism and would produce significant stimulation artifacts making the results difficult to interpret. Optogenetic stimulation is a powerful tool to activate specific neurons through light [58] and has been used to modulate neural activity or restore its relative functions [59,60]. Neural oscillations at various frequency bands can be induced by optogenetic stimulation directly in vivo [61] without any electrical artifact. In the present study, we show that optogenetic stimulation could successfully generate two different types of neural activity in the hippocampal slice by changing the intensity of the laser. One is the suprathreshold spike which is similar to the slow oscillations in the cortex and the hippocampus [34,35,36] and the other is a subthreshold wave both traveling by volume conduction through ephaptic coupling. The stimulation-induced subthreshold waves can propagate across the tissue and are observed simultaneously with the occurrence of suprathreshold spikes. 

In summary, the present study shows that optogenetic stimulation can trigger propagating subthreshold waves in the hippocampus tissue. These subthreshold waves can propagate across a transection, suggesting that electric field coupling is responsible for the propagation. Furthermore, the subthreshold waves can interfere with each other. These results suggest that the neural tissue is wired to allow propagation of waves producing interference patterns with or without synaptic transmission. Although the function of the subthreshold waves in the neural network is unknown, the results described above open the possibility that signal processing of very low amplitude signals could produce interfering patterns below the threshold of neural firing, leading to new level of analog neural computation across neural networks not previously described.

## 4. Methods

### 4.1. Animals

Optopatch transgenic mice [33] were used to conduct the experiments in the present study. The optopatch transgenic mice (stock number: 029679) from the Jackson laboratory were bred with Camk2a-cre transgenic mice (stock number: 005359) to achieve optopatch3 expression on excitatory neurons. Adult male and female mice used for in vitro hippocampal slice studies were at least 60 days old (*p* > 60). All experimental procedures performed in this study followed the NIH animal use guidelines and were approved by the Institutional Animal Care and Use Committee (IACUC) at Case Western Reserve University.

### 4.2. In Vitro Hippocampal Slice Preparation and Recordings

The longitudinal hippocampal slices were prepared for the experiments. Mice of either sex were anesthetized by isoflurane and euthanized by decapitation. Next, the brain was removed rapidly from the skull and was cooled (0–5 °C) in high-sucrose artificial cerebrospinal fluid (Sucrose-aCSF) containing (in mM): sucrose, 220; KCl, 3.0; NaH_2_PO_4_, 1.25; NaHCO_3_, 26; D-glucose, 10; MgSO_4_, 2; CaCl_2_, 2; and bubbled with a 95% O_2_/5% CO_2_ gas mixture. The hippocampus was separated from the brain, cut longitudinally in a thickness of 400 μm, and then incubated in a bubbled normal aCSF at room temperature containing (in mM): NaCl, 125, KCl, 3.75; KH_2_PO_4_, 1.25; D-glucose, 10, NaHCO_3_, 26; MgSO_4_, 2; CaCl_2_, 2. After one hour of incubation, slices were transferred to the interface-recording chamber (Harvard Apparatus) for further experiments. During experiments, the slices were immersed in the lower Ca_2_+ and Mg_2_+ aCSF at room temperature containing (in mM): NaCl, 125, KCl, 3.75; NaH_2_PO_4_, 1.25; D-glucose, 10, NaHCO_3_, 26; MgSO_4_, 1; CaCl_2_, 1. (2R)-amino-5-phosphonovaleric acid (APV, 50 μM) from Sigma-Aldrich and tetrodotoxin (TTX, 1 μM) from Tocris were added in the lower Ca^2+^ and Mg^2+^ aCSF in some experiments. Glass pipette electrodes were placed in the cell layer in the longitudinal hippocampal slice in the interface-recording chamber for signal recordings. All signals were amplified using an Axoclamp-2A microelectrode amplifier (Axon Instruments), low-pass filtered (5 kHz field potentials) with additional amplification (FLA-01, Cygnus Technology), digitized at 20 kHz sampling frequency by using a digitizer (PowerLab 8/35, ADInstruments), and stored in a computer for further analysis.

### 4.3. Optogenetic Stimulation

Optical pulse trains were delivered using a fiber-coupled 473 nm laser system. Stripped and freshly cleaved optical fiber with a 200 µm diameter (0.48 NA, Thorlabs) were placed directly over the hippocampal slice, and the position of the illuminating area was visually adjusted using the stereomicroscope. Optical fibers were connected to a 473 nm DPSS laser (OEM Laser Systems) controlled by an analog voltage input provided by the acquisition system (Powerlab8/35, ADInstruments) to generate a preset optical pulse train. Optical stimulation was applied with 10 ms pulses at 5 Hz, 10 Hz, 20 Hz, and 50 Hz frequencies with a period of 2–4 s. The laser power was controlled by varying the voltage input from the acquisition system through the interfacing software (LabChart 8, ADInstruments) in the range of 0 to 5 volts. 

### 4.4. Statistical Analysis

Statistical analyses were conducted for different comparisons. A one-way ANOVA and post hoc Tukey HSD test were used to compare the speeds and wavelengths of subthreshold waves in different frequency ranges and amplitudes. A *t*-test was used to compare the speed and amplitude difference between suprathreshold spikes and subthreshold waves. A *t*-test was also used to test the amplitude difference between estimated interfering waves and recorded interfering waves in the interference experiments. A paired *t*-test was used to compare the effects of NMDA receptor blocker, APV and tetrodotoxin, TTX. A *p*-value of less than 0.05 was considered statistically significant. All data were represented as mean ± the standard deviation of the mean unless noted.

## Data Availability

All data needed to evaluate the conclusions in the paper are present in the paper. Additional data related to this paper may be requested from the authors.
